# The Role of Serum Free Fatty Acids in Endothelium‐Dependent Microvascular Function

**DOI:** 10.1002/edm2.70031

**Published:** 2025-01-31

**Authors:** Alexander E. Sullivan, Meaghan C. S. Courvan, Aaron W. Aday, David H. Wasserman, Kevin D. Niswender, Emily M. Shardelow, Emily K. Wells, Quinn S. Wells, Matthew S. Freiberg, Joshua A. Beckman

**Affiliations:** ^1^ Division of Cardiovascular Medicine, Department of Medicine Vanderbilt University Medical Center Nashville Tennessee USA; ^2^ Department of Biochemistry University of Colorado Boulder Colorado USA; ^3^ Vanderbilt Translational and Clinical Cardiovascular Research Center, Division of Cardiovascular Medicine Vanderbilt University Medical Center Nashville Tennessee USA; ^4^ Department of Molecular Physiology and Biophysics Vanderbilt University School of Medicine Nashville Tennessee USA; ^5^ Department of Medicine Vanderbilt University Medical Center Nashville Tennessee USA; ^6^ Department of Veteran Affairs Tennessee Valley Healthcare System Nashville Tennessee USA; ^7^ Vanderbilt University Medical Center Program for Metabolic Bone Disorders Nashville Tennessee USA; ^8^ Geriatric Research Education and Clinical Centers (GRECC) Veterans Affairs Tennessee Valley Healthcare System Nashville Tennessee USA; ^9^ Division of Vascular Medicine, Department of Medicine University of Texas Southwestern Dallas Texas USA

**Keywords:** endothelial function, free fatty acid, insulin‐mediated vasodilation, insulin sensitivity, metabolic syndrome

## Abstract

**Background:**

Elevated serum free fatty acid (FFA) concentration is associated with insulin resistance and is a hallmark of metabolic syndrome. A pathological feature of insulin resistance is impaired endothelial function.

**Objective:**

To investigate the effect of FFA reduction with either acipimox, a nicotinic acid derivative that impairs lipolysis, or salsalate, a salicylate that reduces basal and inflammation‐induced lipolysis, on insulin‐mediated endothelium‐dependent vasodilation.

**Methods:**

This was a post hoc, combined analysis of two randomised, double‐blind, placebo‐controlled crossover trials. Sixteen subjects were recruited (6 with metabolic syndrome and 10 controls) and randomised to acipimox 250 mg orally every 6 h for 7 days or placebo. Nineteen subjects were recruited (13 with metabolic syndrome and 6 controls) and randomised to receive salsalate 4.5 g/day for 4 weeks or placebo. The primary outcome was the association between FFA concentration and insulin‐mediated vasodilation, measured by venous‐occlusion strain‐gauge plethysmography at baseline and following FFA modulation with the study drugs.

**Results:**

At baseline, FFA concentration (*R* = −0.35, *p* = 0.043) and insulin sensitivity (HOMA‐IR: *R* = −0.42, *p* = 0.016, Adipo‐IR: *R* = −0.39, *p* = 0.025) predicted insulin‐mediated vasodilation. FFA levels were significantly reduced after drug pretreatment (0.604 vs. 0.491 mmol/L, *p* = 0.036) while insulin levels, insulin sensitivity and inflammatory markers were unchanged. Despite a reduction in circulating FFA with drug therapy, neither insulin‐stimulated vasodilation nor insulin sensitivity improved.

**Conclusions:**

Short‐term reduction of FFA concentration does not improve insulin‐stimulated vasodilation in patients with metabolic syndrome.

**Trial Registration:**

ClinicalTrials.gov identifier: NCT00759291 and NCT00760019 (formerly NCT00762827)

## Graphical abstract

At baseline, free fatty acid concentration and insulin sensitivity predicted insulin‐mediated vasodilation. Acipimox and salsalate reduced free fatty acid concentration, but did not change insulin sensitivity and there was no association between the change in free fatty acids and insulin‐mediated vasodilation.

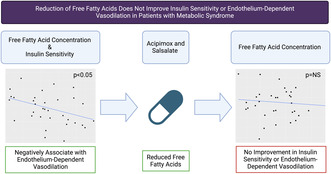

## 1 Introduction

Metabolic syndrome is characterised by progressive insulin resistance, dyslipidaemia and obesity and increases the risk of cardiovascular disease [1]. Disruption of glucose homeostasis due to insulin resistance is associated with increased inflammation, lipolysis and free fatty acid (FFA) efflux [1, 2]. These metabolic changes impair endothelial cell function, initiating atherosclerosis and increasing the risk of ischemic heart disease, stroke and cardiovascular death [3].

Hyperglycaemia, oxidative stress and inflammation have all been mechanistically linked to endothelial dysfunction and studied as potential therapeutic targets to reduce the risk of cardiovascular disease [2, 4, 5, 6, 7, 8, 9]. Long‐term treatments that lower glucose, oxidative stress and inflammation have, however, had limited success in improving endothelial function and seem to be dependent on the mechanism of drug action [9, 10, 11]. For example, in the treatment of hyperglycaemia, glucagon‐like peptide‐1 receptor agonists (GLP‐1RA) have been shown to reduce cardiovascular events and mortality, while dipeptidyl peptidase 4 (DPP4) inhibitors have not shown the same benefits [12, 13].

Similarly, serum FFA are elevated in metabolic syndrome and are thought to be a critical mediator of vascular endothelial dysfunction and adverse cardiovascular events [14, 15]. Acute elevations in FFA levels impair endothelium‐dependent vasodilation, while suppression of lipolysis improves endothelial function [16]. Rapidly lowering serum FFA improves insulin sensitivity, but effects on endothelial function are less well understood [16, 17]. As such, FFA modulation remains a therapeutic target to improve endothelial function in patients with metabolic syndrome.

Acipimox and salsalate are two drugs that have been shown to lower serum FFA concentrations and improve insulin sensitivity. Acipimox is a nicotinic acid derivative that inhibits triglyceride lipase and reduces FFA release. Salsalate, a salicylate, in high doses reduces FFA release and TNF‐α–induced lipolysis and has been found to mitigate the deleterious effects of acute intralipid infusion on endothelial function [18, 19]. Insulin infusion increases capillary blood flow via endothelium‐dependent nitric oxide production; however, the impact of FFA reduction on insulin‐mediated vasodilation is unknown [20]. To better understand the role of FFA modulation on vascular function in those with metabolic syndrome, we conducted a combined, post hoc analysis of two randomised‐controlled trials assessing the effects of acipimox and salsalate on FFA reduction and insulin‐mediated, endothelium‐dependent vasodilation.

## 2 Materials and Methods

### 2.1 Trial Oversight

This report presents the results of two trials (NCT00759291 and NCT00760019 [formerly NCT00762827]) [21, 22] conducted by the authors. The studies were reviewed and approved by the local institutional review board. Written informed consent was obtained from all study participants.

### 2.2 Subject Selection

Volunteers 18 years old and older were recruited by advertisement and from outpatient clinics (Figure S1). Healthy controls were without metabolic syndrome, had a normal physical exam and had a fasting serum glucose < 100 mg/dL. Metabolic syndrome was defined as the presence of > 3 components of the syndrome as defined by the National Cholesterol Education Program (Table S1). Subjects were excluded if they had uncontrolled hypertension (≥ 140/100 mmHg), untreated hyperlipidaemia (low‐density lipoprotein cholesterol > 75th percentile for age), diabetes mellitus, cigarette smoking within 1 year, a history or a physical exam consistent with atherosclerotic disease and renal (creatinine > 1.4 mg/dL) or hepatic dysfunction (alanine aminotransferase concentration > 2‐fold above normal). A full list of inclusion and exclusion criteria for the trials can be found in Table S2.

### 2.3 Study Design

Both studies were randomised, placebo‐controlled, double‐blind crossover trials. All participants underwent a screening evaluation and physical exam to ensure study criteria were met. Treatment adherence was assessed by pill count in both studies.

#### 2.3.1 Acipimox

After ensuring eligibility, participants were randomly allocated to either 7 days of acipimox 250 mg orally every 6 h or a matching placebo by the Investigational Drug Service at Brigham and Women's Hospital. Participants were instructed to take the medication with food to minimise gastrointestinal side effects and to abstain from alcohol and caffeine for 12 h before all subsequent study visits. The final dose was administered at 7 am on the morning of the study visit. Participants then reported in a fasting state, at which time blood profiles and vascular function testing were assessed. After a 4‐week washout period, participants crossed over to receive the other study medication and underwent repeat fasting blood profiles and vascular function testing after 7 days of treatment.

#### 2.3.2 Salsalate

Volunteers who met study eligibility criteria during the screening evaluation were randomly allocated to a 4‐week treatment period of either salsalate 4.5 g/day or matching placebo by the Investigational Drug Service. Salsalate was administered in three 500‐mg Disalcid tablets (Caraco Pharmaceutical Laboratories) and prescribed three times daily. Omeprazole 20 mg daily (Sandoz Inc.) was administered during each treatment period to minimise gastrointestinal side effects. Participants who experienced treatment side effects despite proton pump inhibitor therapy were asked to reduce the daily salsalate dose to 4 g/day, and then 3 g/day if symptoms persisted. The final dose of the study drug was administered at 7 am on the morning of the study visit after the 4‐week treatment period. Participants reported in the fasting state and underwent blood profiles and vascular function testing. After a 4‐week washout period, participants crossed over to the other drug for a 4‐week treatment period, at which time they presented for repeat blood profiles and vascular function testing.

### 2.4 Hyperinsulinaemic–Euglycaemic Clamp

To evaluate insulin sensitivity and insulin‐mediated vasodilatory function within the forearm vasculature, a whole‐body hyperinsulinaemic–euglycaemic clamp was performed via a continuous infusion of insulin at a rate of 80 mU/m^2^/min for a period of a 120 min to attain typical postprandial insulin concentrations. Dextrose 20% solution was infused to maintain a constant glucose concentration of 90 mg/dL. Insulin and glucose levels were assessed from the contralateral cubital vein. Whole‐body insulin sensitivity was determined during the final five steady‐state measurements. Forearm blood flow (FBF) was assessed prior to and during the hyperinsulinaemic clamp at 2 h after steady‐state was achieved.

### 2.5 Forearm Blood Flow

FBF was measured using venous occlusion, mercury‐in‐silastic, strain‐gauge plethysmography, as we have previously described [23, 24]. To perform this measurement, forearm arterial inflow is isolated by (1) excluding hand circulation with a wrist sphygmomanometric cuff inflated to 200 mmHg; and (2) venous outflow using a second sphygmomanometric cuff above the elbow inflated to 40 mmHg for venous occlusion. Forearm arterial inflow, or FBF, is then dependent only on arterial flow. Insulin is a known endothelium‐dependent vasodilator with greater vasodilatory impact in skeletal microvasculature than conduit arteries [25]. FBF can be derived from the rate of change in forearm circumference during cuff inflation. Determination of FBF was the mean of five separate measurements recorded on a Hokanson AI6 physiologic recorder (Hokanson Vascular, Bellevue, WA). During FBF assessment, the vascular laboratory was kept quiet, dimly lit and temperature controlled at 23°C.

### 2.6 Laboratory Measurements

Participant laboratory assessments included fasting glucose, complete blood count, lipid profile, glycosylated haemoglobin, renal function and hepatic function (Sunquest Laboratory, Tucson, AZ). Salicylate levels were assessed on the Olympus AU640 analyser (Beckman Coulter Ltd.). Plasma insulin was assessed using an Immunoenzymatic Sandwich Assay (Beckman Coulter Inc., Brea, CA). The concentration of nonesterified FFAs was determined using an in vitro enzymatic colorimetric assay on a Hitachi 917 analyzer (Roche Diagnostics) with reagents from Wako Chemicals USA (Richmond, VA). High‐sensitivity C‐reactive protein (hsCRP) was measured using an immunoturbidimetric assay on a Hitachi 917 analyzer (Roche Diagnostics, Indianapolis, IN) using reagents and calibrators from DiaSorin (Stillwater, MN). Tumour necrosis factor (TNF)‐α receptor 2 was measured using an ELISA (R&D Systems, Minneapolis, MN). Myeloperoxidase (MPO) was assessed using ELISA (Alpco Diagnostics, Salem, NH). All assays were performed by a core laboratory certified by the National Heart, Lunch, and Blood Institute, Centers for Disease Control, and Prevention Lipid Standardization Program by personnel blinded to treatment and metabolic syndrome status.

### 2.7 Endpoints and Biomarkers of Interest

Endothelium‐dependent vasodilation was assessed both at rest and during a hyperinsulinaemic clamp. The vasodilatory response was measured as the difference in FBF during the hyperinsulinaemic clamp compared to resting FBF (also called insulin‐stimulated FBF change). The Homeostatic Model Assessment of Insulin Resistance (HOMA‐IR) was calculated from fasting insulin and glucose levels (fasting plasma insulin, mIU/L) × (fasting plasma glucose, mg/dL)/405 [26]. The adipose tissue insulin resistance index (Adipo‐IR [mmol × pmol/L]) was calculated by multiplying the fasting FFA concentration (mmol/L) by the fasting insulin concentration (pmol/L) [27]. The M index was calculated by dividing the glucose infusion rate during the last hour of the insulin clamp by the mean insulin concentration [28]. The primary endpoint of interest was the association between FFA levels and the change in FBF with the hyperinsulinaemic clamp.

### 2.8 Statistical Analysis

Baseline characteristics were presented as mean and standard deviation or count and percentages as appropriate. Comparisons of baseline characteristics were performed between healthy controls and those with metabolic syndrome using the Wilcoxon rank‐sum test for continuous variables and the chi‐squared test for categorical data. Association between variables of interest and endothelial‐dependent vasodilation was assessed using a Pearson correlation coefficient. Multivariable linear regression was used to control for baseline differences, including study drug assignment (acipimox or salsalate) and metabolic syndrome. Only participants who attended all study visits and underwent endothelial function assessment were included in the analysis. All analyses were performed using R (version 4.2.2).

## 3 Results

### 3.1 Patient Characteristics

Subject recruitment is shown in Figure S1. A total of 35 participants completed the acipimox study protocol and 30 participants completed the salsalate study protocol. Primary results from these studies have previously been published [21, 22]. Sixteen participants from the acipimox arm and 19 participants from the salsalate arm had complete FBF data and were eligible for this subgroup analysis. In both arms, participants with metabolic syndrome were older, with higher baseline systolic blood pressure, diastolic blood pressure, triglycerides, fasting blood glucose, fasting insulin level and insulin sensitivity assessed by HOMA‐IR (Table 1). Participants with metabolic syndrome also had a higher body mass index (BMI) in the salsalate arm. There was no difference in serum creatinine nor high‐density lipoprotein cholesterol (HDL‐C).

**TABLE 1:** Baseline characteristics of patients in the two trials.

	Acipimox			Salsalate		
	Metabolic syndrome (*n* = 6)	Healthy (*n* = 10)	*p*	Metabolic syndrome (*n* = 13)	Healthy (*n* = 6)	*p*
Age, years	63.8 (4.5)	49.9 (5.2)	< 0.01	57.1 (9.6)	36.3 (9.3)	< 0.01
Sex						
Female	4 (66.7%)	6 (60.0%)	1.00	6 (46.2%)	3 (50.0%)	1.00
Male	2 (33.3%)	4 (40.0%)		7 (53.8%)	3 (50.0%)	
Race						
White	5 (83.3%)	9 (90.0%)	0.32	9 (69.2%)	5 (83.3%)	0.72
African American	0 (0%)	1 (10.0%)		3 (23.1%)	1 (16.7%)	
Hispanic	1 (16.7%)	0 (0%)		0 (0%)	0 (0%)	
Other	0 (0%)	0 (0%)		1 (7.7%)	0 (0%)	
Body mass index (km/m^2^)	30.9 (1.5)	27.1 (6.0)	0.55	33.9 (4.7)	25.5 (2.5)	< 0.01
Waist circumference (inches)	41.3 (3.4)	37.0 (6.1)	0.12	44.2 (5.3)	35.1 (4.0)	< 0.01
Systolic blood pressure (mmHg)	161 (31.4)	120 (6.3)	0.02	133 (15.3)	114 (9.9)	0.01
Diastolic blood pressure (mmHg)	89.7 (11.9)	74.0 (6.9)	0.03	83.0 (9.7)	71.8 (6.5)	0.02
Creatinine (mg/dL)	0.932 (0.2)	0.787 (0.2)	0.25	0.935 (0.2)	0.917 (0.3)	0.60
HDL‐C (mg/dL)	53.3 (17.8)	63.5 (14.8)	0.21	41.5 (14.5)	45.2 (9.5)	0.33
Triglycerides (mg/dL)	187 (72.4)	86.2 (36.2)	0.01	187 (114.0)	61.5 (14.8)	< 0.01
Baseline blood glucose (mg/dL)	86.3 (18.8)	80.7 (5.9)	0.05	98.6 (7.1)	83.3 (8.0)	< 0.01
Baseline insulin level (μU/mL)	15.9 (9.5)	3.48 (1.4)	< 0.01	10.8 (5.2)^a^	4.27 (2.3)	0.01
HOMA‐IR	3.28 (2.0)	0.681 (0.2)	< 0.01	2.66 (1.4)^a^	0.878 (0.5)	0.01

*Note:* Data presented as mean (standard deviation) unless otherwise noted. Wilcoxon rank‐sum tests were used to obtain *p* values for continuous variables. Chi‐squared tests were used to obtain *p* values for categorical variables.

Abbreviations: HDL‐C, high‐density lipoprotein; HOMA‐IR, Homeostatic Model Assessment of Insulin Resistance.

^a^ Indicates 1 missing value.

### 3.2 FBF After Placebo Pretreatment

**FIGURE 1:**
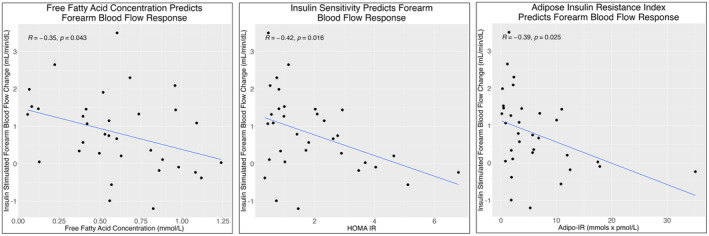
Free fatty acid concentration (left), insulin sensitivity (middle) and adipose tissue insulin resistance (right) associate with forearm blood flow response to insulin stimulation after placebo pretreatment.

At baseline, one participant had supraphysiologic FBF that was felt to be an outlier resulting from measurement error and was excluded from the analysis. Baseline FBF in all participants following placebo pretreatment was 2.20 ± 1.04 mL/100 g/min and increased to 3.02 ± 1.22 mL/100 g/min at peak insulin stimulation (Table 2). The mean increase in the vasodilatory response was 0.826 ± 1.05 mL/100 g/min. Insulin sensitivity, assessed by HOMA‐IR (*R* = −0.42, *p* = 0.016) and Adipo‐IR (*R* = −0.39, *p* = 0.025), negatively correlated with vasodilatory response (Figure 1). Similar results were seen using the M index (Figure S2). Baseline FFA concentration negatively correlated with the vasodilatory response to insulin stimulation (*R* = −0.35, *p* = 0.043, Figure 1).

**TABLE 2:** Forearm blood flow at rest and with insulin stimulation after pretreatment with placebo, acipimox or salsalate.

	Placebo (*n* = 34)	Drug (*n* = 34)	*p* *
Mean baseline arterial flow (mL/100 g/min)	2.20 (1.0)	2.29 (1.0)	0.59
Mean insulin‐stimulated arterial flow (mL/100 g/min)	3.02 (1.2)	3.06 (1.3)	0.85
Mean arterial flow response to hyperinsulinaemia Clamp (mL/100 g/min)	0.826 (1.1)	0.768 (0.8)	0.80

*Note:* All data are shown as mean (standard deviation). No interaction effect of assigned drug (acipimox vs. salsalate) in multivariable linear regression.

* *p* values were obtained from paired Wilcoxon rank‐sum tests.

### 3.3 FFA, Insulin Sensitivity and Inflammation Modulation by Drug Pretreatment

**FIGURE 2:**
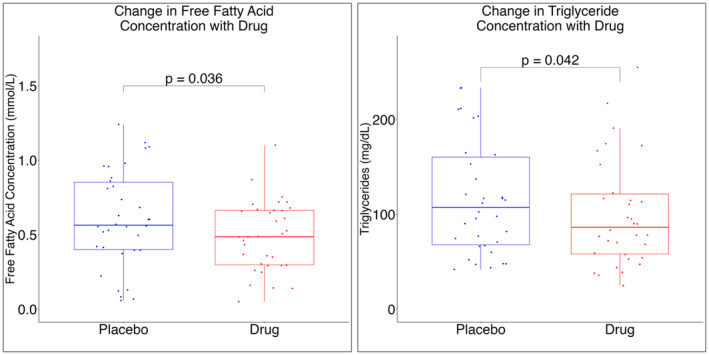
Free fatty acid (A) and triglyceride (B) concentrations were reduced by acipimox and salsalate.

There was a significant reduction in serum FFA concentration (0.604 vs. 0.491 mmol/L, *p* = 0.036) and serum triglyceride concentration (127 vs. 111 mg/dL, *p* = 0.0416) after drug treatment (Figure 2). Reduction in serum FFA concentrations during hyperinsulinaemia was not significantly different after drug exposure (*p* = 0.207), although the absolute serum FFA concentration during hyperinsulinaemia (0.090 vs. 0.068 mmol/L, *p* = 0.045) was significantly reduced by drug pretreatment (Figure S3). Other markers of inflammation and insulin resistance did not change significantly with either drug or placebo pretreatment (Table 3).

**TABLE 3:** Effect of placebo or drug pretreatment on markers of lipid metabolism, inflammation and insulin sensitivity.

	Placebo (*n* = 34)	Drug (*n* = 34)	*p*
Total cholesterol (mg/dL)^a^	165 (27.9)	162 (30.6)	0.32
HDL‐C (mg/dL)	46.9 (13.4)	47.6 (13.8)	0.90
hsCRP (mg/L)	3.08 (4.2)	2.40 (2.8)	0.11
TNFR2 (pg/mL)	2600 (1520.0)	2500 (1490.0)	0.86
MPO (mg/mL)^b^	34.5 (80.8)	27.1 (27.0)	0.87
Baseline insulin concentration (pmol/L)^c^	8.59 (6.9)	8.50 (5.9)	0.48
HOMA IR^c^	1.92 (1.6)	1.82 (1.3)	0.28
Adipo‐IR^c^	5.95 (7.1)	4.75 (5.3)	0.35
M index^d^	4.54 (1.9)	4.60 (2.79)	0.55
Post‐insulin clamp FFA concentration (mmol/L)^a^	0.090 (0.06)	0.068 (0.04)	0.05

*Note:* All data are shown as mean (standard deviation). *p* values were obtained from a paired Wilcoxon ranked sum test.

Abbreviations: Adipo‐IR, adipose tissue insulin resistance index; HDL‐C, high‐density lipoprotein cholesterol; HOMA IR, Homeostatic Model Assessment of Insulin Resistance; hsCRP, High‐sensitivity C‐reactive protein; FFA, Free Fatty Acid; MPO, myeloperoxidase; TNFR‐2, tumour necrosis factor receptor‐2.

^a^ Number missing: Data only available from the salsalate trial.

^b^ Number missing: 3 from both placebo and drug.

^c^ Number missing: 1 from both placebo and drug.

^d^ Number missing: 10 from placebo, 8 from drug.

### 3.4 FBF After Drug Pretreatment

**FIGURE 3:**
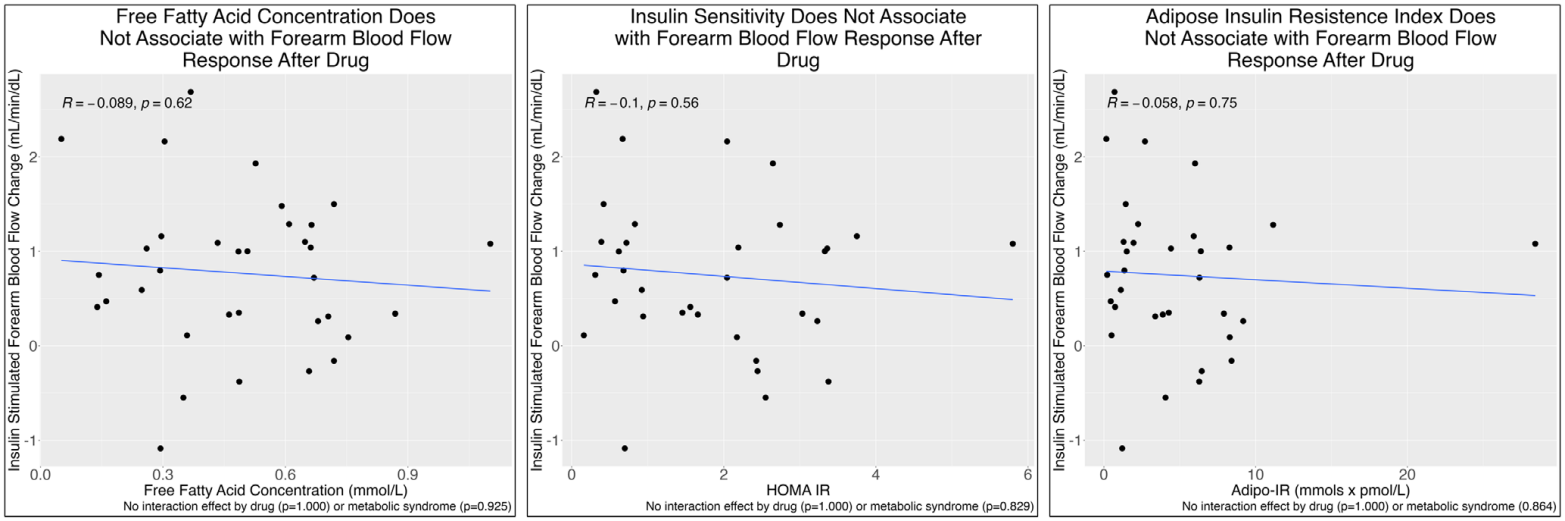
Free fatty acid concentration (left), insulin sensitivity (middle) and adipose tissue insulin resistance (right) do not associate with forearm blood flow response to insulin stimulation after drug treatment.

There was no change in resting FBF (2.20 vs. 2.29 mL/100 g/min, *p* = 0.590), peak FBF after insulin stimulation (3.02 vs. 3.06 mL/100 g/min, *p* = 0.851) or vasodilatory response to hyperinsulinaemia (0.826 vs. 0.768 mL/100 g/min, *p* = 0.800) between placebo and drug treatment (Table 2). Results were similar even after controlling for differences in drug exposure (acipimox vs. salsalate) and metabolic syndrome. Sensitivity analyses considered acipimox and salsalate independently, yielding similar results. After drug, neither FFA concentration (*R* = −0.089, *p* = 0.620), HOMA‐IR (*R* = −0.1, *p* = 0.560), Adipo‐IR (*R* = −0.058, *p* = 0.750) nor M index (*R* = 0.21, *p* = 0.300) correlated with vasodilatory response to hyperinsulinaemic clamp (Figure 3; Figure S2). This relationship was altered neither by the pretreatment drug (acipimox or salsalate) nor the presence of metabolic syndrome. There was also no relationship between the change in FFA concentration following drug vs. placebo treatment with the change in resting or peak insulin‐stimulated FBF (Figure S4). Similarly, changes in FFA levels did not associate with the change in vasodilatory response to insulin stimulation following placebo vs. drug treatment. There was no relationship between change in HOMA‐IR and Adipo‐IR with change in resting FBF, peak insulin‐stimulated FBF or vasodilatory response to insulin stimulation between placebo and drug (Figures S5 and S6). There was no relationship between FFA reduction from rest to hyperinsulinaemia and the vasodilatory response to hyperinsulinaemia, regardless of placebo or drug pretreatment (Figure S3).

### 3.5 Sensitivity Analyses

Two sensitivity analyses were conducted to ensure consistency of the above results. The first sensitivity analysis was conducted to assess the differential effects of the two drugs (acipimox and salsalate) and a second analysis considered only patients with metabolic syndrome. There were no significant differences from the findings presented above, although power was a significant limitation in these analyses.

## 4 Discussion

Among participants with metabolic syndrome and healthy controls, serum FFA concentration prior to drug pretreatment was negatively associated with insulin‐mediated, endothelium‐dependent vasodilation. Significant reductions in serum FFA concentration were observed after pretreatment with either acipimox or salsalate, while other markers of inflammation and insulin sensitivity were unchanged. Post‐treatment endothelium‐dependent vasodilation was unchanged, and post‐treatment FFA concentrations no longer associated with insulin‐mediated endothelium‐dependent vasodilation. This suggests that FFA may represent a marker of metabolic disease that associates with endothelial function under fasting conditions rather than having a direct impact on endothelial function.

While acipimox and salsalate were both initially thought to improve insulin sensitivity, data supporting this is inconsistent. Salsalate reduces fasting glucose by decreasing insulin clearance and thereby increasing serum insulin levels by up to 10% without a significant change in insulin sensitivity [29, 30]. Salsalate, therefore, acts more similarly to a secretagogue than a sensitising agent. Similarly, while some have demonstrated improved insulin sensitivity with acipimox [31], data from multiple other studies have found no effect of acipimox on insulin sensitivity [32, 33]. Given this discordance in the literature, we took a unifying approach to interrogate the relationship between FFA reduction, insulin sensitivity and endothelial function. Much of the data linking FFA levels to insulin sensitivity has been drawn from investigations where FFA levels were increased well beyond physiological levels with infusion of intralipid and heparin [19, 34]. However, the reduction of chronically elevated levels, as in our study, has not been found to improve insulin sensitivity or endothelial function [17, 21, 33]. Thus, our own work, resting on a significant amount of prior investigation by others over several decades, links the reductions in FFA mediated by both salsalate and acipimox to no improvement in insulin sensitivity and to no improvement in insulin‐mediated arteriolar recruitment.

Impaired endothelial function is an inciting event in the atherosclerotic cascade and is associated with increased cardiovascular events and mortality in those with and without established cardiovascular disease [15, 35]. As such, there has been significant interest in interventions that restore endothelial function in participants with cardiovascular disease. Hyperglycaemia, reactive oxygen species and inflammation have all been mechanistically linked to impaired eNOS and attenuated NO bioavailability, but modifying these targets pharmacologically has not consistently reduced cardiovascular events [4, 9, 10, 11, 24]. Intensive blood glucose control with a combination of oral therapies and insulin increased the risk of cardiovascular death, while more modest reductions with GLPR1 agonists and SGLT2 inhibitors have shown cardioprotective properties [8, 10, 13]. Similarly, acute supraphysiologic infusion of vitamin C mitigates endothelial dysfunction caused by acute hyperglycaemia, but oral antioxidant vitamin supplementation does not reduce cardiovascular events or death [4, 11]. Modulating inflammation with canakinumab or colchicine reduces recurrent cardiovascular events, although similar results were not seen with methotrexate [7, 9]. Our results add to these observations and suggest that FFA reduction, at least via mechanisms of action of acipimox and salsalate, is ineffective. The preponderance of evidence suggests that the underlying drug mechanisms have a greater impact on endothelial function than merely the reduction in the metabolic target.

To date, no large‐scale randomised trial has demonstrated a significant improvement in endothelial function and cardiovascular events with FFA reduction. FFA may not directly modulate endothelial function; rather, FFA may serve as a metabolic biomarker of other adverse metabolic changes that drive endothelial dysfunction. While acute elevations in FFA worsen endothelial function, reducing chronic elevations has not effectively improved endothelial function. There are several potential explanations. Acute elevations in FFA levels with intralipid and heparin infusion achieve much higher FFA levels than chronic insulin resistance–induced elevations, and therefore may have a different mechanistic effect on endothelial function. FFA levels in these studies reached as high as 1.3 mmol/L, nearly twice that seen in both chronic obesity and the present cohort [21, 33, 34]. Both acute and chronically elevated FFA levels have been shown to induce endothelial dysfunction through several mechanisms that impair insulin signalling and NO synthesis while also increasing oxidative stress, inflammation, activation of the renal‐angiotensin system (RAS) and endothelial cell apoptosis. Despite these similarities, reducing FFA levels after acute versus chronic exposure does not lead to the same improvement in endothelial function [21, 22, 33]. Acute reduction of chronically elevated FFA levels does not break the maladaptive cycle concurrently driven by inflammation, RAS activation and endothelial apoptosis, nor does it address FFA‐associated mishandling of small low‐density lipoprotein cholesterol (LDL‐C) [36].

FFA may, therefore, be a biomarker of the pro‐atherosclerotic milieu that accompanies insulin resistance rather than a primary driver of this dysfunction. Our data suggest that targeting FFA alone neglects the multiple maladaptive metabolic mechanisms by which the vascular‐insulin resistance homeostasis is been perturbed in metabolic syndrome [37]. By utilising acipimox and salsalate, we were able to isolate the role of FFA reduction on insulin sensitivity and endothelial function and observe no other drug‐specific effects. As expected, pretreatment with acipimox and salsalate significantly reduced FFA levels but had no effect on insulin resistance, regardless of whether it was assessed utilising insulin‐glucose homeostasis (HOMA‐IR) or insulin‐FFA homeostasis (Adipo‐IR). Similarly, FFA reduction did not improve insulin‐mediated arteriolar recruitment. These observations provide further evidence that FFA may serve as a biomarker of maladaptive metabolism and that FFA‐targeted interventions will not improve insulin resistance or endothelial dysfunction.

The present findings are concordant with prior work modulating FFA levels. While prior studies have shown that acute elevation in FFA concentrations with intralipid and heparin infusion worsens endothelial function in healthy adults, FFA reduction has not been shown to have the opposite effect [16, 38]. A single dose of acipimox results in a short‐term 74% reduction in plasma FFA concentration, but no improvement in endothelial‐dependent vasodilation in the microvasculature [16]. Similarly, in conduit arteries, while acute elevations in FFA with intralipid diminish brachial artery flow‐mediated vasodilation, no improvement has been shown with FFA reduction [21, 22]. Fenofibrate has been shown to improve endothelium‐dependent vasodilation, but this occurs in association with a significant reduction in triglyceride levels without any effect on FFA concentration [39]. While the role of FFA and impaired NO bioavailability has been the subject of several investigations, it is the balance of vasodilators and vasoconstrictors, including angiotensin II, endothelin‐1, thromboxane A2 and prostaglandin H_2_, that ultimately determines endothelial vasodilatory function [14]. Further investigation into the relationship between FFA and elaborated vasoconstrictors may provide insights into the limited improvement in endothelial function noted with prior interventions. Furthermore, in contrast to intralipid infusion, FFA levels undulate during periods of feeding and fasting in those with chronic insulin resistance and obesity [40]. The role these prandial shifts play, rather than merely the magnitude of FFA elevation, in endothelial dysfunction is incompletely understood.

Our study has several limitations. This was a post hoc analysis of two similar and simultaneous randomised trials. Only 54% of participants had complete FBF data, and so our data may not detect a true difference in endothelial function, although our findings are consistent with our previous reports. The study population size may have limited our ability to detect a difference in insulin sensitivity following drug treatment. Previous studies with acipimox suggest a sample size of 72 would be required to demonstrate a statistically significant difference [33]. Our data suggest that, with the current effect size, 10,000 participants in a crossover design would be required to have enough statistical power to demonstrate a difference in insulin‐stimulated vasodilation. This study is not feasible, and therefore, this is an unavoidable limitation given the minimal improvement in endothelial function with drug therapy. Our analysis was designed to assess the role of FFA modulation on insulin‐stimulated vasodilation irrespective of drug. Acipimox or salsalate act via two different mechanisms, and there may be unaccounted‐for effects related to this. A sensitivity analysis was conducted considering the data separately for each drug, which yielded results similar to the pooled analysis. Furthermore, both drugs were shown in separate analyses to reduce FFA concentrations without any effect on insulin sensitivity or inflammation. It is therefore unlikely that our results are confounded by differential effects of the drugs. While the two drugs were administered for different treatment periods, these time periods were selected based on prior data demonstrating the treatment time needed for effective FFA reduction [21, 22]. Finally, our analysis focuses on the effects of plasma FFA, and it remains unknown what is occurring at the cellular level.

## 5 Conclusion

In summary, data from this post hoc analysis of two simultaneously conducted, double‐blinded, randomised, placebo‐controlled crossover design trials show that FFA concentrations are reduced with acipimox and salsalate. While FFA levels correlate with endothelial function at baseline, FFA reduction does not improve endothelial‐dependent vasodilation. Given the growing metabolic syndrome epidemic in the United States, studies that evaluate a multi‐targeted approach to ameliorate endothelial dysfunction are needed.

## Author Contributions

J.A.B. conceived, planned, and executed the presented idea. A.E.S., M.C.S.C., and Q.S.W. defined the statistical analysis plan and conducted the formal analysis. All authors contributed to the conceptualizing, writing, and editing of the manuscript. All authors have read and given approval of the final version to be published, have agreed on the journal to which the article has been submitted and agreed to be accountable and responsible for all aspects of the work.

## Disclosure

A.W.A.: Dr. Aday reports receiving consulting fees from Aeglea outside of the current work; J.A.B.: Consulting: JanOne; DSMC for Janssen and Novartis. The other authors have nothing to report.

## Conflicts of Interest

The authors declare no conflicts of interest.

## Supporting information


**Figure S1.** Consort diagram showing patient recruitment and attrition during the two trials.
**Figure S2.** Insulin sensitivity (by M index) associates with forearm blood flow response to insulin stimulation after placebo (left), but not after drug (right) pretreatment.
**Figure S3.** Drug pretreatment did not significantly alter the change in FFA levels after hyperinsulinaemic clamp, but absolute FFA concentration was lower at hyperinsulinaemic peak with drug pretreatment compared to placebo. There was no correlation between FFA change during hyperinsulinaemia and forearm blood flow response with either drug or placebo.
**Figure S4.** Change in free fatty acid concentration after drug treatment does not associate with change in baseline forearm blood flow (left), peak forearm blood flow (middle) or forearm blood flow response to insulin stimulation (right).
**Figure S5.** Change in homeostatic model of assessment of insulin resistance after drug treatment does not associate with baseline forearm blood flow (left), peak forearm blood flow (middle) or forearm blood flow response to insulin stimulation (right).
**Figure S6.** Change in adipose tissue insulin resistance index after drug treatment does not associate with baseline forearm blood flow (left), peak forearm blood flow (middle) or forearm blood flow response to insulin stimulation (right).
**Table S1.** Components of metabolic syndrome.
**Table S2.** Trial inclusion and exclusion criteria.

## Data Availability

Restrictions apply to the availability of some or all data generated or analysed during this study to preserve patient confidentiality or because they were used under licence. The corresponding author will, on request, detail the restrictions and any conditions under which access to some data may be provided.
