# Supplementary material for: The Role of Serum Free Fatty Acids in Endothelium‐Dependent Microvascular Function
Source: Endocrinol Diabetes Metab. 2025 Jan 31;8(2):e70031. doi: 10.1002/edm2.70031 (PMC11784902; doi:10.1002/edm2.70031)
Supplement: Supplementary file 1 — Figure S1. Consort diagram showing patient recruitment and attrition during the two trials. Figure S2. Insulin sensitivity (by M index) associates with forearm blood flow response to insulin stimulation after placebo (left), but not after drug (right) pretreatment. Figure S3. Drug pretreatment did not significantly alter the change in FFA levels after hyperinsulinaemic clamp, but absolute FFA concentration was lower at hyperinsulinaemic peak with drug pretreatment compared to placebo. There was no correlation between FFA change during hyperinsulinaemia and forearm blood flow response with either drug or placebo. Figure S4. Change in free fatty acid concentration after drug treatment does not associate with change in baseline forearm blood flow (left), peak forearm blood flow (middle) or forearm blood flow response to insulin stimulation (right). Figure S5. Change in homeostatic model of assessment of insulin resistance after drug treatment does not associate with baseline forearm blood flow (left), peak forearm blood flow (middle) or forearm blood flow response to insulin stimulation (right). Figure S6. Change in adipose tissue insulin resistance index after drug treatment does not associate with baseline forearm blood flow (left), peak forearm blood flow (middle) or forearm blood flow response to insulin stimulation (right). Table S1. Components of metabolic syndrome. Table S2. Trial inclusion and exclusion criteria. [file EDM2-8-e70031-s001.zip › EDM2_70031_f_Supplemental Data_EDM.docx]

Supplemental Table 1

| **Components of the Metabolic Syndrome** | |
| --- | --- |
| **Risk Factor** | **Criteria** |
| Abdominal obesity | Waist circumference |
| Men | > 101.6 cm |
| Women | > 88.9 cm |
| Triglycerides | ≥ 150 mg/dL |
| High-density lipoprotein cholesterol |  |
| Men | < 40 mg/dL |
| Women | < 50 mg/dL |
| Blood pressure | ≥ 130/≥ 85 mmHg |
| Fasting glucose | ≥ 110 mg/dL |

Adapted from Executive Summary of the Third Report of the National Cholesterol Education Program (NCEP) Expert Panel on Detection, Evaluation, and Treatment of High Blood Cholesterol in Adults (Adult Treatment Panel III): Expert Panel on Detection, Evaluation, and Treatment of High Blood Cholesterol in Adults. *JAMA*. 2001;285:2486-2497.

Supplemental Table 2

|  | Acipimox Arm | Salsalate Arm |
| --- | --- | --- |
| Inclusion Criteria | - Patients 18-years or older | - Patients 18-years or older |
| Exclusion Criteria | - Uncontrolled hypertension (≥140/100 mm Hg) - Untreated hyperlipidemia (low-density lipoprotein cholesterol > 75^th^ percentile for age) - Diabetes mellitus - Cigarette smoking within 1 year - History of atherosclerotic disease or a physical exam consistent with atherosclerotic disease - Renal insufficiency (creatinine > 1.4 mg/dL) - Blood cell dyscrasia - Hepatic Dysfunction (alanine aminotransferase concentration > 2-fold above normal) | - Uncontrolled hypertension (≥140/100 mm Hg) - Untreated hyperlipidemia (low-density lipoprotein cholesterol > 75^th^ percentile for age) - Diabetes mellitus - Cigarette smoking within 1 year - Neuropathy requiring medication - Nephropathy (>300mg/24-hour urine albumin or serum creatinine > 1.4 mg/dL) - Hypersensitivity to salicylates - Increased bleeding risk (including thrombocytopenia, hemorrhagic stroke within 1 year, or concomitant use of anticoagulation) - Ingestion of >5 alcoholic beverages/week - Hepatic Dysfunction (alanine aminotransferase concentration > 2-fold above normal) - Chronic inflammatory disorder (eg, rheumatoid arthritis or systemic lupus erythematosus) - Active malignancy |
| Other Considerations | - Individuals receiving statin therapy were required to discontinue the drug two week prior to randomization through the duration of the trial |  |


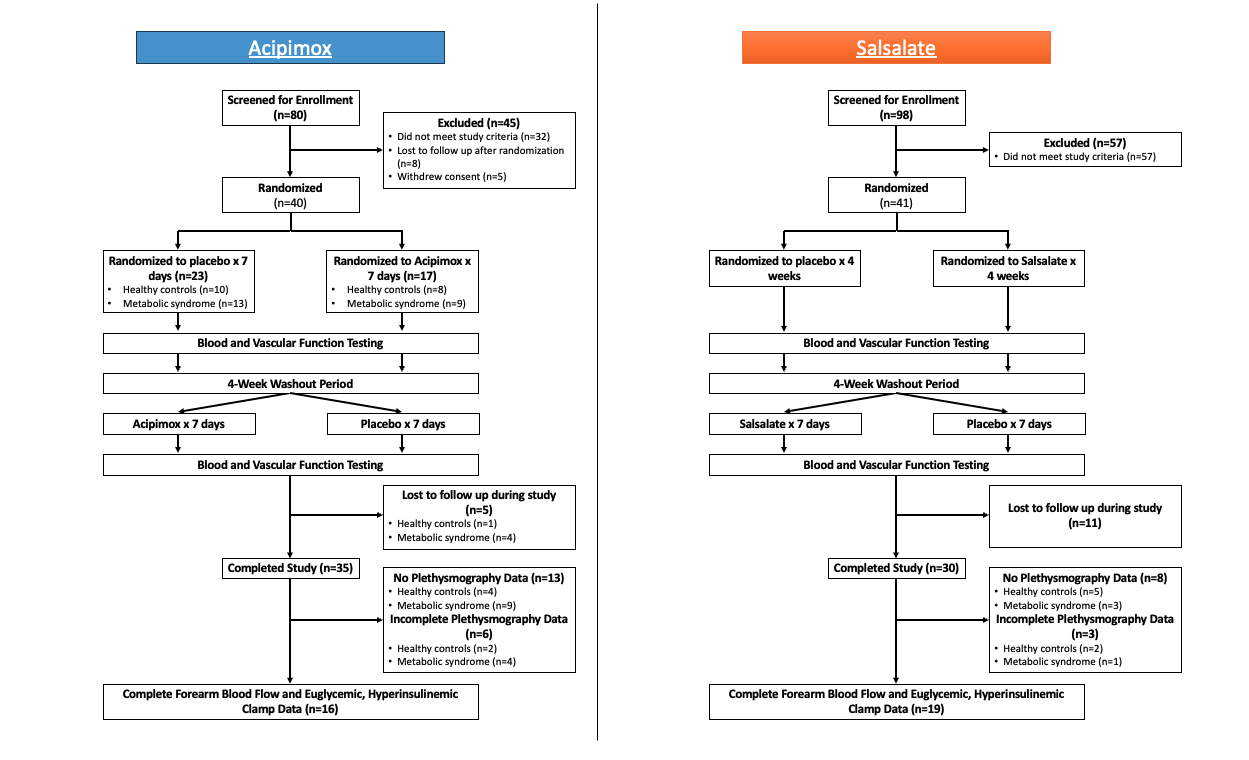
Supplemental Figure 1: Consort diagram showing patient recruitment and attrition during the two trials

Supplemental Figure 2: Insulin Sensitivity (by M index) Associates with Forearm Blood Flow Response to Insulin Stimulation After Placebo (left), but not Drug (right) Pretreatment.


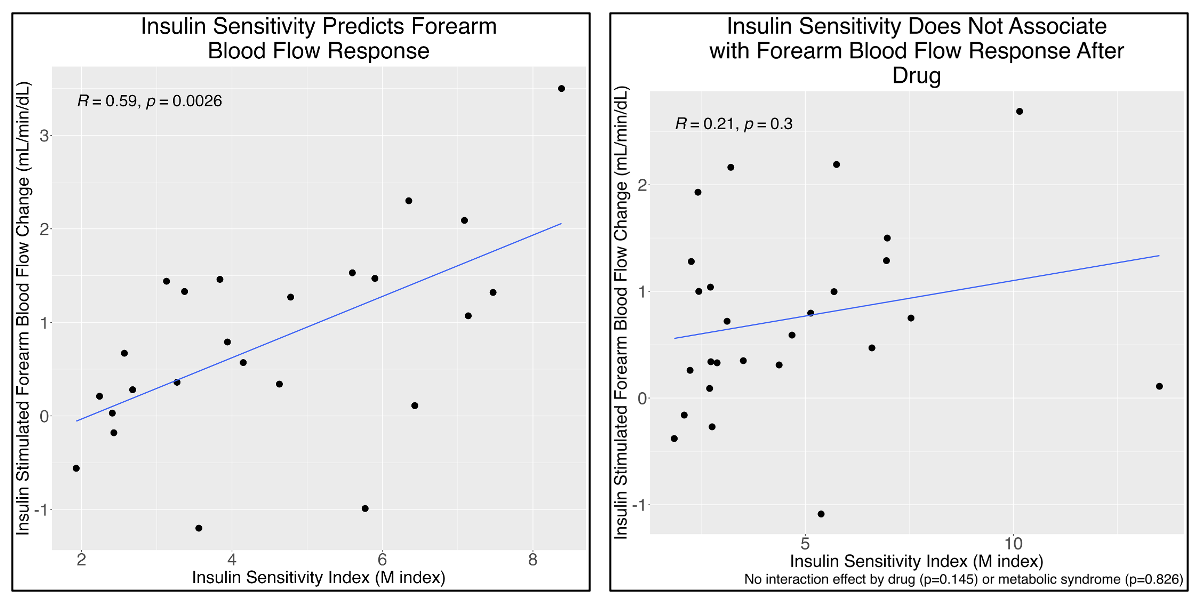


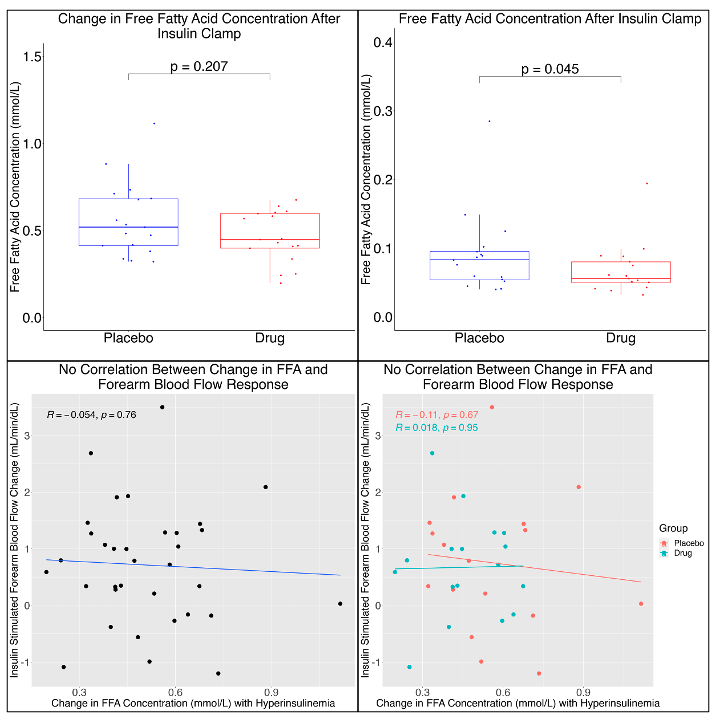
Supplemental Figure 3: Drug pretreatment did not significantly alter the change in FFA levels after hyperinsulinemic clamp, but absolute FFA concentration was lower at hyperinsulinemic peak with drug pretreatment compared to placebo. There was no correlation between FFA change during hyperinsulinemia and forearm blood flow response with either drug or placebo.

FFA: free fatty acids

Supplemental Figure 4: Change in Free Fatty Acid Concentration After Drug Treatment Does Not Associate with Change in Baseline Forearm Blood Flow (left), Peak Forearm Blood Flow (middle), or Forearm Blood Flow Response to Insulin Stimulation (right).


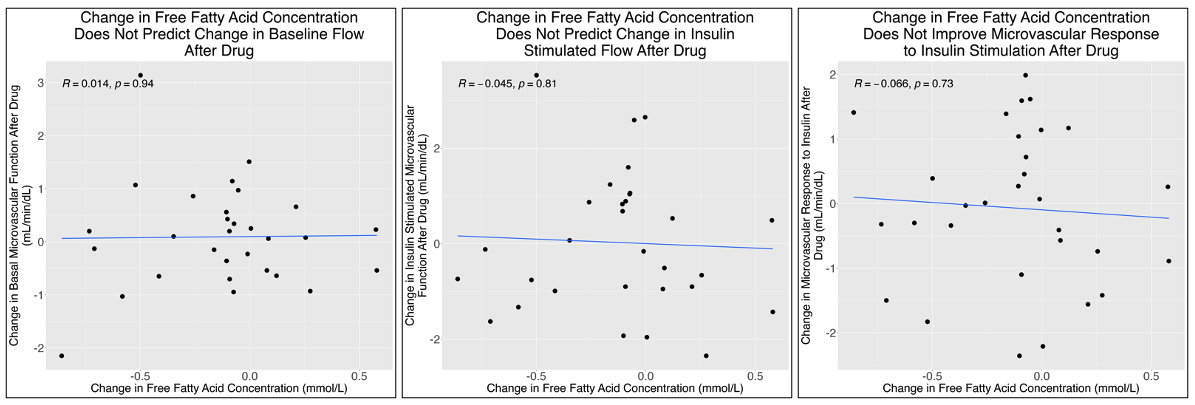


Supplemental Figure 5: Change in Homeostatic Model of Assessment of Insulin Resistance After Drug Treatment Does Not Associate with Baseline Forearm Blood Flow (left), Peak Forearm Blood Flow (middle), or Forearm Blood Flow Response to Insulin Stimulation (right).


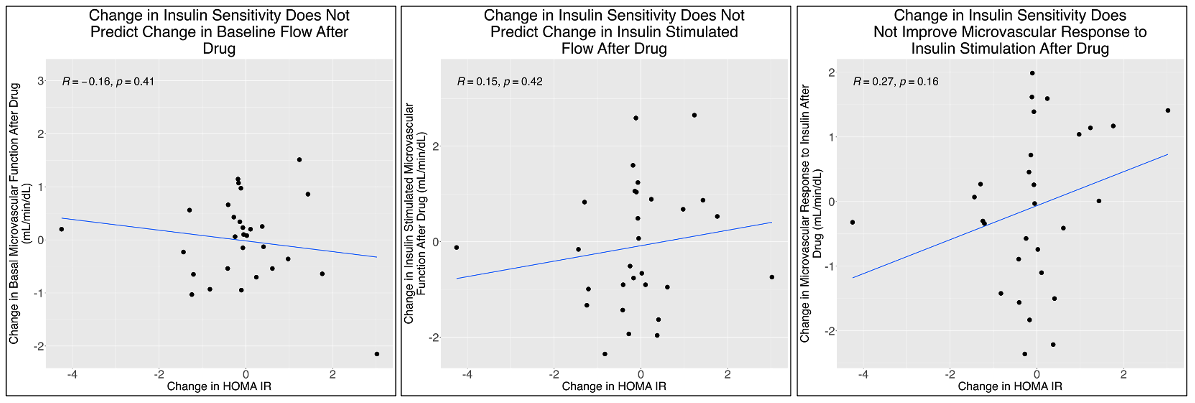


HOMA-IR: Homeostatic Model Assessment of Insulin Resistance

Supplemental Figure 6: Change in Adipose Tissue Insulin Resistance Index After Drug Treatment Does Not Associate with Baseline Forearm Blood Flow (left), Peak Forearm Blood Flow (middle), or Forearm Blood Flow Response to Insulin Stimulation (right).


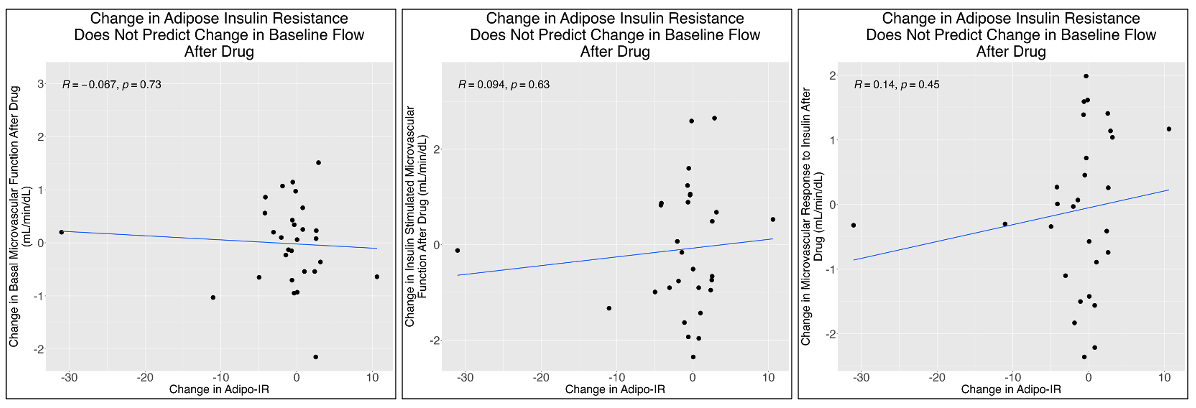


Adipo-IR: adipose tissue insulin resistance index
